# Transdermal delivery of traditional Chinese medicine patch vs. NSAIDs patch for alleviating inflammation and relieving pain for early-stage knee osteoarthritis: a retrospective case control study

**DOI:** 10.3389/fphar.2025.1549883

**Published:** 2025-04-03

**Authors:** Shoufeng Wang, Jingtao Guan, Wanran Gong, Bingbo Bao

**Affiliations:** ^1^ Department of Orthopedics, The First Affiliated Hospital of Jiamusi University, Jiamusi, China; ^2^ Department of Orthopedics, Shanghai Sixth People’s Hospital affiliated to Shanghai Jiao Tong University School of Medicine, Shanghai, China

**Keywords:** early-stage knee osteoarthritis, traditional Chinese medicine, NSAIDs, transdermal, patch

## Abstract

**Background:**

The effects of transdermal delivery of traditional Chinese medicine (TCM) patch for early-stage knee osteoarthritis (EKOA) is unclear.

**Objective:**

This study is aimed to compare the therapeutic effects of a type of TCM topical drug-Xiaotong patch with NSAIDs topical drug-flurbiprofen patch to treat EKOA.

**Methods:**

This retrospective case control study included 42 EKOA patients from October 2023 to September 2024. Patients were divided into Xiaotong patch group and flurbiprofen patch group. The baseline characteristics, such as demographic and epidemiological information were collected. The main outcome measured was the alteration in Visual Analogue Scale (VAS) score following treatment. The secondary outcomes included inflammatory markers, like cytokine tumor necrosis factor-alpha (TNF-α) and erythrocyte sedimentation rate (ESR).

**Results:**

The 42 EKOA patients were divided into two groups averagely. They received the transdermal patch therapy daily for 14 days. The primary outcome-pain assessment based on VAS score showed a prominent decrease in both groups compared with the values before treatment (P < 0.05). There were no significant differences between groups after treatment (P > 0.05). For the secondary outcomes, TNF-α and ESR were included for evaluating the pre- and post-treatment findings. The results also indicated the inflammatory conditions were alleviated by transdermal delivery of drugs from TCM patch or NSAIDs patch. Similarly, the data showed a comparable anti-inflammatory effect between groups (P > 0.05).

**Conclusion:**

The TCM transdermal patch exerts a similar effect on the EKOA in the aspects of pain relief and regulating inflammation for a short-term treatment as NSAIDs patch. It may provide an alternative for clinical management of EKOA.

## Introduction

Knee osteoarthritis (KOA) is a prevalent disease among elderly population, with classic symptoms of knee pain, joint deformity and dysfunction. The main pathological manifestations are persistent inflammation, fibrosis, wear and exfoliation of knee cartilage, accompanied by synovial hyperemia and edema and osteophyte formation (Lotz and Caramés, 2011). With the increasing aging of society, the social and family burden caused by the disease will be further aggravated in the next few decades (Katz et al., 2021). The early-stage KOA (EKOA) is characterized by the small extent of tissue destruction of the bone and subchondral bone (Liew et al., 2023). Proper treatment can help to slow, stop or reverse disease progression (Arendt et al., 2014). Compared with moderate and advanced KOA, the diagnosis and treatment of EKOA focuses on timely diagnosis, pain relief and function restoration. Meanwhile, special attention should be paid to tissue injuries such as knee cartilage and subchondral bone to promote their repair to delay, prevent or even reverse the progress of EKOA.

It is not hard to diagnose EKOA. While, the proper management of EKOA requires better care and attention with some advances in the recent years. The primary care and therapy for EKOA patients include health education and self-management, weight control, exercise therapy, physical therapy and the use of knee assistive devices and other basic treatment. In addition to the basic treatment, medication, such as oral and topical drug use, is a significant aspect of the EKOA treatment (Bannuru et al., 2019). Topical NSAIDs application is used as the first-line administration of EKOA. Topical NSAIDs are available in different formulations, such as gels, creams, sprays or patch formulations, to be applied to the affected area. A real-world meta-analysis showed that topical NSAIDs were more successful in diminishing pain and augmenting function than acetaminophen, but not as effective as oral NSAIDs (Zeng et al., 2021). For patients with persistent knee pain or EKOA who do not respond to topical drugs, oral NSAIDs are recommended as first-line oral drugs for pain relief (Wolff et al., 2021). Opioid analgesics are classified as second-line oral drugs (Goodwin et al., 2009; Jiang et al., 2024). Oral NSAIDs have possible negative impacts on the gastrointestinal, cardiovascular, and renal systems (Pelletier et al., 2016). OARSI, ACR and other societies have recommended that patients with gastrointestinal reactions during the use of NSAIDs should use selective COX-2 inhibitors, or non-selective NSAIDs combined with proton pump inhibitors (Nakata et al., 2018; Laine et al., 2008; Lanas, 2007). NSAIDs are not recommended in patients with stage IV and V of chronic kidney disease, and NSAIDs in patients with stage III need to be evaluated (Baker and Perazella, 2020). NSAIDs can increase the risk of cardiovascular adverse events, and cardiovascular risk should be assessed before use (Varga et al., 2017). Therefore, it is quite practical and important to evaluate lasting and more effective topical drugs with good controlled release pattern, high safety and effectiveness to modulate pathological conditions.

With better understanding on traditional herbal medicine, external use of traditional Chinese medicine (TCM), which includes Chinese patent herbal paste, plaster or ointment, has proved useful and good therapeutic outcomes (Wang et al., 2012a; Wang et al., 2012b). Chinese medicine components in the patch or other formations with controlled release style can be absorbed through the local skin and directly act on the joint and its surrounding tissues, exert local analgesic, anti-inflammatory and microcirculation improvement effects, and help to reduce the swelling of the soft tissues around the joint (Lin et al., 2022). TCM for external use should be selected according to different symptoms and signs. Therefore, in this study, we aim to compare the therapeutic effects of a type of TCM topical drug-Xiaotong patch with NSAIDs topical drug-flurbiprofen to treat EKOA. We comprehensively evaluate basic information, and epidemiological characteristics of these patients with EKOA and their sensory function and inflammatory conditions before and after treatment. Hopefully, we will provide preliminary evidence for alternative therapy on EKOA treatment in the clinical work.

## Materials and methods

### General information

This study involved a retrospective review of clinical data from 42 patients diagnosed with EKOA in the outpatient department between October 2023 to September 2024. Based on different treatment methods, the patients were divided into Xiaotong patch group (21 cases) and flurbiprofen patch group (21 cases).

In Xiaotong patch group, there were 10 male and 11 female patients, with ages ranging from 45 to 68 years (54.71 ± 6.84 years). The disease duration is 7.00 (5.00, 9.00) months. The Visual Analogue Scale (VAS) score was 6.00 (6.00.7.00). In flurbiprofen patch group, there were 12 male and nine female patients, with ages ranging from 41 to 69 years (54.95 ± 8.35 years). The disease duration is 10.80 (7.00, 14.50) months. The median VAS score was 6.00 (6.00.7.00). No significant variations were found between two groups regarding gender, age, disease duration, and other baseline characteristics (P > 0.05), demonstrating their comparability.

### Inclusion criteria

1. Meet the diagnostic criteria for EKOA.
2. Experience knee pain within the past month, with pain occurring for more than half of the day.
3. Age between 40 and 79 years.
4. In accordance with the ethical principles outlined in the Declaration of Helsinki.

### Exclusion criteria

1. History of drug allergies.
2. Abnormal liver or kidney function.
3. Previous history of severe knee joint injuries.
4. NSAIDs treatment within the past month.

### Treatment methods

For the treatment of Xiaotong patch group, Xiaotong patch (Tibet Linzhi Qizheng) was applied externally on the affected area. Xiaotong patch should be applied one patch/day, and the treatment should last for 14 days continuously. Patients in flurbiprofen patch group were treated with flurbiprofen patch (Beijing Tide Pharmaceutical) to treat the affected area one patch/day, with continuous treatment for 14 days. All patients received health education and proper exercise therapy along with the medication.

### Observation indicators

VAS Score: Pain intensity was assessed using a 10-point scale, where scores of 1–3 (mild pain), 4–6 (moderate pain), 7–9 (severe pain), and 10 (extreme pain).

Erythrocyte Sedimentation Rate (ESR): ESR quantifies the speed at which red blood cells fall to the bottom of a test tube containing whole blood.

Tumor Necrosis Factor Alpha (TNF-α):TNF-α levels in serum were assessed before and after topical administration in both groups.

### Statistical methods

Data were statistically processed using SPSS 29.0 software. For measurement data, Shapiro-Wilk normality test was performed. Intergroup differences were analyzed with the t-test or Wilcoxon signed-rank test (when it did not meet the parametric test conditions). Normally distributed variables were summarized using the mean and standard deviation ( $\bar{x}$ ± s). Data that did not follow normal distribution were expressed as median (25th percentile, 75th percentile). When the data exhibited a normal distribution, paired t-tests were conducted to assess the differences in scores before and after treatment within each group. For data failing to meet normal distribution criteria, the Wilcoxon signed-rank test was employed for comparisons. P < 0.05 was considered statistically significant.

## Results

### Intergroup comparison of general information

There were no significant differences between Xiaotong patch group and flurbiprofen patch group in gender, residence, smoking, alcohol, diabetes, hypertension, and Kellgren-Lawrence grade (Table 1).

**TABLE 1:** Intergroup comparison of general information.

	Xiaotong group	Flurbiprofen group	χ2	P
Gender				
Male	10	12	0.382	0.537
Female	11	9		
Residence				
Local	10	8	0.389	0.533
Provincial	11	13		
Smoking				
Yes	7	9	0.404	0.525
No	14	12		
Alcohol				
Yes	10	15	2.471	0.116
No	11	6		
Diabetes				
Yes	5	7	0.467	0.495
No	16	14		
Hypertension				
Yes	7	7	-	1.000
No	14	14		
Kellgren-Lawrence grade				
0	4	4	0.117	0.943
I	9	8		
II	8	9		

Intergroup comparison of VAS, score before and after treatment.

### Intergroup comparison of VAS score before and after treatment

The Shapiro-Wilk normality test revealed P values of 0.084 and 0.021 for pre-treatment VAS scores, and 0.024 and 0.002 for post-treatment VAS scores in the Xiaotong and Flurbiprofen groups, respectively. As these results indicated non-normal distribution (P < 0.05), we employed Wilcoxon signed-rank tests for the statistical analysis.

Prior to treatment, there were no significant differences in VAS score between groups. After treatment, both the Xiaotong and flurbiprofen groups exhibited a significant decrease in VAS score. However, the post-treatment VAS score did not differ significantly between the Xiaotong and flurbiprofen groups (P > 0.05, Table 2).

**TABLE 2:** Intergroup comparison of VAS score before and after treatment.

	Number of participants (N = 42)	VAS score M(QL,QU)	
		Before treatment	After treatment
Xiaotong group	21	6.00 (6.00, 7.00)	3.00 (2.00, 3.00)*
Flurbiprofen group	21	6.00 (6.00, 7.00)	3.00 (3.00, 4.00)*
Z		−0.382	−0.822
P		0.702	0.411

VAS, is used to value pain intensity. Values are expressed as median (lower quartile, upper quartile). *P < 0.05 indicates significant difference compared to pre-treatment values within the same group. Z and P values represent between-group comparisons at each time point.

### Intergroup comparison of ESR before and after treatment

After performing the Shapiro-Wilk normality test, the P values for pre-treatment ESR were 0.086 and 0.251 in the Xiaotong and Flurbiprofen groups, respectively. The P values for post-treatment ESR were 0.138 and 0.095 in the Xiaotong and Flurbiprofen groups, respectively. Since P > 0.05 indicated normal distribution, we employed t-tests for statistical analysis.

Initial ESR assessments revealed no significant differences between groups prior to treatment. After treatment, both groups experienced a significant reduction in ESR, but this value was slightly higher in Xiaotong patch group than flurbiprofen patch group (P > 0.05, Table 3).

**TABLE 3:** Intergroup comparison of ESR before and after treatment.

	Number of participants (N = 42)	ESR (mm/h)	
		Before treatment	After treatment
Xiaotong group	21	27.05 ± 12.749	23.90 ± 10.261*
Flurbiprofen group	21	24.81 ± 12.335	21.43 ± 10.078*
t		0.578	0.789
P		0.566	0.435

Data are presented as mean ± standard deviation. N: number of participants. ESR, serves as a widely used inflammatory marker. *P < 0.05 is used to show comparison with pre-treatment values within the same group. The t and P values represent between-group comparisons at each time point.

### Intergroup comparison of TNF-α, before and after treatment.

The Shapiro-Wilk normality test revealed P values of 0.223 and 0.072 for pre-treatment TNF-α, and 0.514 and 0.061 for post-treatment TNF-α, in the Xiaotong and Flurbiprofen groups, respectively. As P > 0.05 indicated normal distribution, t-tests were used for statistical analysis.

Before treatment, there were no significant differences in ESR, between groups. After treatment, there was a significant decrease in ESR, in both groups and this value was slightly lower in Xiaotong patch group than flurbiprofen patch group (P > 0.05, Table 4).

**TABLE 4:** Intergroup comparison of TNF-α before and after treatment.

	Number of participants (N = 42)	TNF-α (pg/mL)	
		Before treatment	After treatment
Xiaotong group	21	7.81 ± 4.364	5.95 ± 3.018*
Flurbiprofen group	21	8.01 ± 5.779	7.12 ± 4.897*
t		−0.127	−0.933
P		0.900	0.356

Data are presented as mean ± standard deviation. N: number of participants. TNF-α, is a key inflammatory marker used to evaluate inflammatory responses. TNF-α, plays a central role in mediating inflammation, being an early-response pro-inflammatory cytokine that initiates and regulates the inflammatory cascade. *P < 0.05 is used to show comparison with pre-treatment values within the same group. The t and P values represent between-group comparisons at each time point.

### Intergroup comparison of mild allergy and edema of the joint after treatment

The rate of mild allergic reactions did not differ significantly between groups (Table 5), and the allergic reaction was only slight rashes that disappear soon after patch removal. The edema of the joint was alleviated in both groups. The clinical manifestation showed that microcirculation was also improved within the period of treatment.

**TABLE 5:** Intergroup comparison of mild allergy of the joint after treatment.

	Xiaotong group	Flurbiprofen group	χ2	P
Mild Allergy				
Yes	2	2	-	1
No	19	19		

## Discussion

At present, the defining elements of EKOA include K-L grading based on X-ray imaging, cartilage and subchondral bone marrow injury from MRI imaging, symptoms and sign (Pan et al., 2024; Li et al., 2023). One of the many important elements that define EKOA is K-L grading system based on anterior-lateral X-rays of the knee in the weight-bearing (standing) position. In this study, we evaluated the EKOA condition before treatment, primarily by K-L grade based on X-rays, in addition to VAS score, to show the morphological changes of the affected knee in each patient.

The main characteristics of EKOA in the Chinese guideline for diagnosis and non-surgical treatment of early-stage knee osteoarthritis (2024 edition) include (1) Recurrent knee pain over 1 month; (2) Age ≥ 40 years; (3) Joint stiffness ≤ 30 min, pain when squatting and standing up, or going up and down stairs; (4) Bone frictional sounds (sensations) and joint space tenderness; (5) Weight-bearing (standing) X-rays show no significant changes in the joints, or osteophytes and/or suspected narrowing of the joint space; (6) MRI shows localized or diffuse cartilage injury (range ≤ 75%), or subchondral bone marrow edema in any area (range ≥ 25%). EKOA can be diagnosed when the diagnostic criteria 1 + 2 + 3 + 4 + 5 or 1 + 6 are met. Here in this study, we adopted the previous criteria.

EKOA can be diagnosed based on the patient’s history, symptoms, physical examination, and imaging and is featured by chronic strain. It is necessary to know the patient’s age, occupational habits, exercise habits, height, weight, *etc.* Age, obesity or being overweight, and being female are risk factors for EKOA (Liu et al., 2015; Ren et al., 2020). As for the age limit of EKOA patients, there is no clear age setting in domestic and foreign literature at present, and most of the articles defining EKOA patients are over 40 years old (Tang et al., 2016). A systematic review has shown that occupational groups that require long-term weight-bearing are more likely to develop EKOA than sedentary occupations (Ezzat and Li, 2014). Therefore, we included these factors into account in this study to better characterize the main features of EKOA patients.

In addition, we collected some demographic and epidemiological information, such as residence, smoking, alcohol, hypertension and diabetes. For patients living in rural areas, they are prone to developing osteoarthritis due to large amount of farm work and heavy load on the lower limbs compared with local patients in the suburb (Tang et al., 2016). Smoking and alcohol abuse are two primary causes to increase the likeliness of developing gout and synovitis (To et al., 2021; Zhang et al., 2015). Alcohol abuse increases the metabolic burden of the liver and thus produces more metabolites of lactic acid, which leads to soreness after retention in the joints (Liu et al., 2022; Kc et al., 2015). Meanwhile, smoking usually increases the chances of irritation of the synovial area and thus causes congestion, soreness and swelling in the knee joint with early-stage osteoarthritis (Amin et al., 2007). Hypertension and diabetes are susceptibility factors for osteoarthritis (Alenazi et al., 2023; Yang et al., 2024). They may cause arteriosclerosis, hardening of blood vessels, joint stenosis, poor elasticity, slowed blood flow, and inability to keep up with blood oxygen. This not only affects the brain, heart, kidneys and liver, but also influences the blood flow in the joints, and consequently compromises the metabolism and overall use of the articular cartilage surface. The arteriosclerosis occurs throughout the body and affects multiple organs including the bone, thereby reducing the absorption and metabolism of effective bone components. No significant differences were noticed in the factors mentioned above in this study and we basically ruled out the impacts of these factors on the therapeutic outcomes between two groups.

After confirming the diagnosis of EKOA, the proper treatment for this disease, is very important to control the progression and pain relief. Health education comes first as it is the foundation of most chronic diseases. The ways of health education include lectures, outpatient education, media (audio-visual, graphic), social tools and so on. The contents of health education include the mechanism of EKOA pain, the prognosis of the disease, and the risk factors for disease progression to guide patients to manage their lifestyle and change bad living and working habits, such as avoiding running, jumping and squatting for a long time, and reducing or avoiding climbing stairs and mountains (Bowden et al., 2020). Also, it is vital to utilize some physical and exercise therapies. The exercise therapy can help to relieve the pain of EKOA, improve joint motion, increase muscle strength around the knee joint, improve knee stability and proprioception, and delay the disease process (Bannuru et al., 2019; Skou et al., 2018). The physical therapy can help to improve local blood circulation, reduce inflammation, and relieve joint pain and stiffness. The physical therapy includes heat therapy, hydrotherapy, mud bath and other treatment (Shen et al., 2021; Ma et al., 2022; Fraioli et al., 2018). In this study, we encouraged the patients in both groups to follow the health education and accept exercise therapy along with proper medication.

In addition to the basic treatment, the anti-inflammatory medication, both in topical and oral ways, are primary treatment for reducing pain and cartilage degeneration in the EKOA patients. With regards to pharmaceutical drug treatment, NSAIDs and acetaminophen serve as two primary drugs for treating KOA. However, there are several common adverse effects including gastrointestinal, thromboembolic and neural problems (Sohail et al., 2023; McCrae et al., 2018; Vonkeman and van de Laar, 2010). By learning further on KOA, an increasing number of new topical drug delivery criteria and systems are now developed, to make up for the weakness of traditional dosage forms, improve the drug bioavailability and considerably decrease side effects of these drugs (Shentu et al., 2024). Transdermal administration offers a non-invasive method for delivering drugs, effectively bypassing the drawbacks of gastrointestinal irritation and the first-pass metabolism associated with oral intake. Additionally, it provides the benefit of sustained and continuous drug release. Key advantages include enhanced patient compliance, ease of administration, and the ability for patients to self-administer their medications conveniently (Jeong et al., 2021). In this study, we compared a type of TCM topical drug patch with a typical type of NSAIDs, flurbiprofen hydrogel patch to evaluate their anti-inflammatory and analgesic effects in a transdermal drug delivery method tropically for EKOA patients.

The NSAIDs patch used in this study is a well-known drug-flurbiprofen hydrogel patch, and can regulate the inflammation and relieve pain well (Tomatsu et al., 2022). For the TCM topical drug patch used in this study, Xiaotong patch is a classic Tibetan medicine prescription. It overcomes the problems in traditional patches, such as short bioavailability time and insufficient efficacy of the drug. The main ingredient of the Xiaotong patch includes Lamiophlomis, Curcuma longa and other natural Tibetan medicine. Its full components are confidential but is primarily characterized by iridoids, flavone, phenylethanoid glycoside and others. These bioactive components play a major role in anti-inflammation, pain relief and anti-oxidative stress. The bioactive substances in Xiaotong patch can affect the knee joint by entering the human circulation at a constant and sustained rate, avoiding the first pass effect in the liver, improving bioavailability and reducing adverse reactions in a transdermal delivery style. The research showed that some of the major components could penetrate the skin by increasing the hydration level of stratum corneum cells and enlarging the medicine storage space. As for pain relief, Xiaotong patch has a good multi-target analgesic effect. It can stimulate the C-fiber discharge of skin nerve endings in the innervated area, inhibit peripheral inflammatory pain sensitization, and exert a joint effect on the peripheral and central nervous systems. We evaluated the VAS score in both groups before and after treatment. It was reduced considerably after topical use of the TCM Xiaotong patch or NSAIDs flurbiprofen patch. The pain relief is the primary outcome factor for evaluating the therapeutic effect on EKOA. It helps the patients to gain confidence and restart normal functional life and work. Although oral medication exerts a similar or sometimes even better effect on the joint pain, the side effects on the gastrointestinal system are not negligible. Useful transdermal drug delivery therapy is an ideal alternative for EKOA treatment.

Apart from the pain relief, it is also vital to take the inflammation and oxidative stress of the bone and joint system into consideration. The Xiaotong patch also reduces inflammation in the bone joint effectively by regulating NF-κB signaling pathway in the macrophage and reducing cytokine expression levels. TNF-α is a very significant pro-inflammatory factor. Reducing the expression level of TNF-α contributes to easing the progression of EKOA. The matrix metalloproteinases were considerably increased in synovium of the knee joint. TNF-α can activate NF-κB signaling pathway, harm the cartilage tissues, modulate the synthetic and secretory process of pro-inflammatory factors, and inhibit cartilage regeneration (Molnar et al., 2021). In addition, the ESR is another important parameter for evaluation of the inflammatory condition in the human body. It can be reflection of the systemic inflammation in plenty of diseases, including degenerative and rheumatoid osteoarthritis, and infectious diseases like pneumonia (Hanada et al., 2016). We compared the serum level of ESR before and after treatment in two groups to reflect anti-inflammatory effects of the drugs systemically. Inflammation may influence muscle strength in patients with KOA. Thus, the ESR level may serve as reflection of the inflammatory and muscle quality in EKOA patients, as a potential indicator for motor function. In this study, we evaluated the TNF-α and ESR expression levels in the serum before and after topical drug therapy from both groups to show the different extents of these two drugs on modulating the inflammation in the systemic level. These two markers were similarly downregulated in the two groups after treatment with Xiaotong patch or flurbiprofen patch. Although there were no significant differences between groups before or after treatment, the overall downregulation extent was quite comparable in Xiaotong patch group and flurbiprofen patch group. In addition, the edema of the joint was also alleviated in both groups. The clinical manifestation showed that microcirculation was also improved within the period of treatment. The findings indicate that for EKOA, short-term use of either patch by transdermal delivery can well alleviate systemic and joint inflammation status. The biocompatibility of both patches was generally fine, with only 2 cases of mild allergy of rashes in each group. The patients regained normal skin appearance and limb function after removal of the medication. No systemic allergy or other sides effects were found. The findings were consistent to the previous knowledge, showing relatively good safety of the TCM Xiaotong patch in treating EKOA by short-term application.

However, current evidence on the efficacy of TCM for knee osteoarthritis KOA remains controversial, with studies showing varying levels of effectiveness and safety profiles across different traditional treatment approaches. More rigorous studies are needed to eliminate potential biases and enhance the quality of evidence (Hou et al., 2015).

Several limitations of this study should be acknowledged. First, the relatively small sample size (n = 42) may limit the generalizability of our findings. Secondly, the 14-day follow-up period was insufficient to evaluate long-term efficacy and safety outcomes. Thirdly, the retrospective design of our study may introduce potential biases affecting result validity. Fourthly, the lack of double-blinding could introduce observer bias, particularly in the subjective assessment of VAS scores. Patients’ physical activity levels or exercise therapy might also influence treatment outcomes. Finally, while TNF-α and ESR provide valuable inflammatory insights, the evaluation of additional inflammatory markers could offer a more comprehensive understanding of the inflammatory changes. It is necessary to conduct a large-scale, prospective, double-blind randomized controlled trial with extended follow-up duration, comprehensive inflammation status assessment and exercise management in the future to provide more robust evidence for comparative efficacy and safety of Xiaotong patch *versus* NSAIDs patch in EKOA treatment.

## Conclusion

In this study, we evaluate a few parameters and generally found a comparable effect in the control of pain, inflammation and oxidative stress between classical TCM Xiaotong patch and NSAIDs flurbiprofen patch, in a relatively short-term evaluation for treating EKOA. The information provides us with potential choices and alternatives in treating early-stage osteoarthritis. Longer term of evaluation and assessment on the biosafety and effectiveness is also important, especially based on a large case-control study to improve the clinical work and expand the indication of the transdermal patch treatment.

## Data Availability

The original contributions presented in the study are included in the article/supplementary material, further inquiries can be directed to the corresponding author.
